# Supplementary material for: Glacial Runoff Promotes Deep Burial of Sulfur Cycling-Associated Microorganisms in Marine Sediments
Source: Front Microbiol. 2019 Nov 7;10:2558. doi: 10.3389/fmicb.2019.02558 (PMC6853847; doi:10.3389/fmicb.2019.02558)
Supplement: Supplementary file 1 [file Data_Sheet_1.zip › Supplementary files S1-3/Description of Supplementary Files S1-S3.docx]

**Description of Supplementary Files S1-S3**

**Supplementary File S1**

Reference alignment of DsrAB sequences used for phylogenetic placement and classification of *dsB*-OTU sequences. This file contains all aligned 1292 DsrAB sequences from a reference database (Müller *et al.* 2015) and novel DsrAB sequences from diverse candidate phyla (Anantharaman et al., 2017; Hausmann et al., 2018; Parks et al., 2017).

**Supplementary File S2**

The updated DsrAB reference tree file used for phylogenetic placement and classification of *dsB*-OTU sequences.

**Supplementary File S3**

Reference taxonomy file containing the taxomic information of all DsrAB sequences in the updated DsrAB reference tree. The *first* column indicates the name of the sequence, the other three columns indicate the individual taxomic levels:

- The *second* column indicates the ‘DsrAB-type’.
- The *third* column indicates the ‘genus’ (within the reductive archaeal-type DsrAB), the ‘phylum_class’ (within the oxidative bacterial-type DsrAB), or the ‘DsrAB-cluster’ (within the reductive bacterial-type DsrAB).
- The *fourth* column indicates the ‘DsrAB-family’ (taxonomic families based on known isolates and uncultured family-level lineages within the reductive bacterial-type DsrAB).

**References**

Anantharaman, K., Hausmann, B., Jungbluth, S. P., Kantor, R. S., Lavy, A., Warren, L. A., et al. (2018). Expanded diversity of microbial groups that shape

the dissimilatory sulfur cycle. ISME J. 12, 1715–1728.

Hausmann, B., Pelikan, C., Herbold, C. W., Köstlbacher, S., Albertsen, M., Eichorst, S. A., et al. (2018). Peatland Acidobacteria with a dissimilatory sulfur metabolism. ISME J. 12, 1729–1742.

Müller, A. L., Kjeldsen, K. U., Rattei, T., Pester, M., and Loy, A. (2015). Phylogenetic and environmental diversity of DsrAB-type dissimilatory (bi)sulfite reductases. ISME J. 9, 1152–1165.

Parks, D. H., Rinke, C., Chuvochina, M., Chaumeil, P.-A., Woodcroft, B. J., Evans,

P. N., et al. (2017). Recovery of nearly 8,000 metagenome-assembled genomes substantially expands the tree of life. Nat Microbiol 2, 1533–1542.
