# Supplementary material for: Glacial Runoff Promotes Deep Burial of Sulfur Cycling-Associated Microorganisms in Marine Sediments
Source: Front Microbiol. 2019 Nov 7;10:2558. doi: 10.3389/fmicb.2019.02558 (PMC6853847; doi:10.3389/fmicb.2019.02558)
Supplement: Supplementary file 2 [file Data_Sheet_2.PDF]

# Glacial runoff promotes deep burial of sulfur cycling-associated microorganisms in marine sediments

## *Supplementary Material*

Claus Pelikan, Marion Jaussi, Kenneth Wasmund, Marit-Solveig Seidenkrantz, Christof Pearce, Zou Zou Anna Kuzyk, Craig W. Herbold, Hans Røy, Kasper Urup Kjeldsen, and Alexander Loy

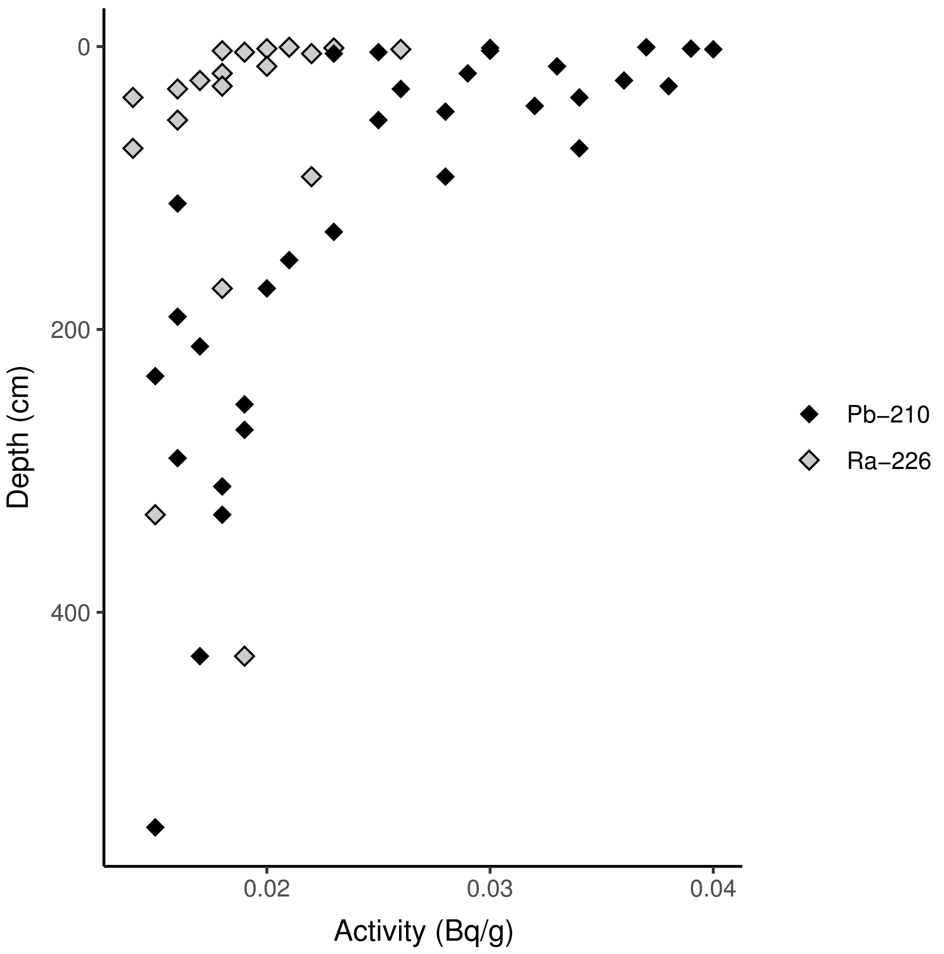


**Supplementary Figure S1. Activity of $^{210}$Pb (black) and $^{226}$Ra (grey) at station St8-GI.**

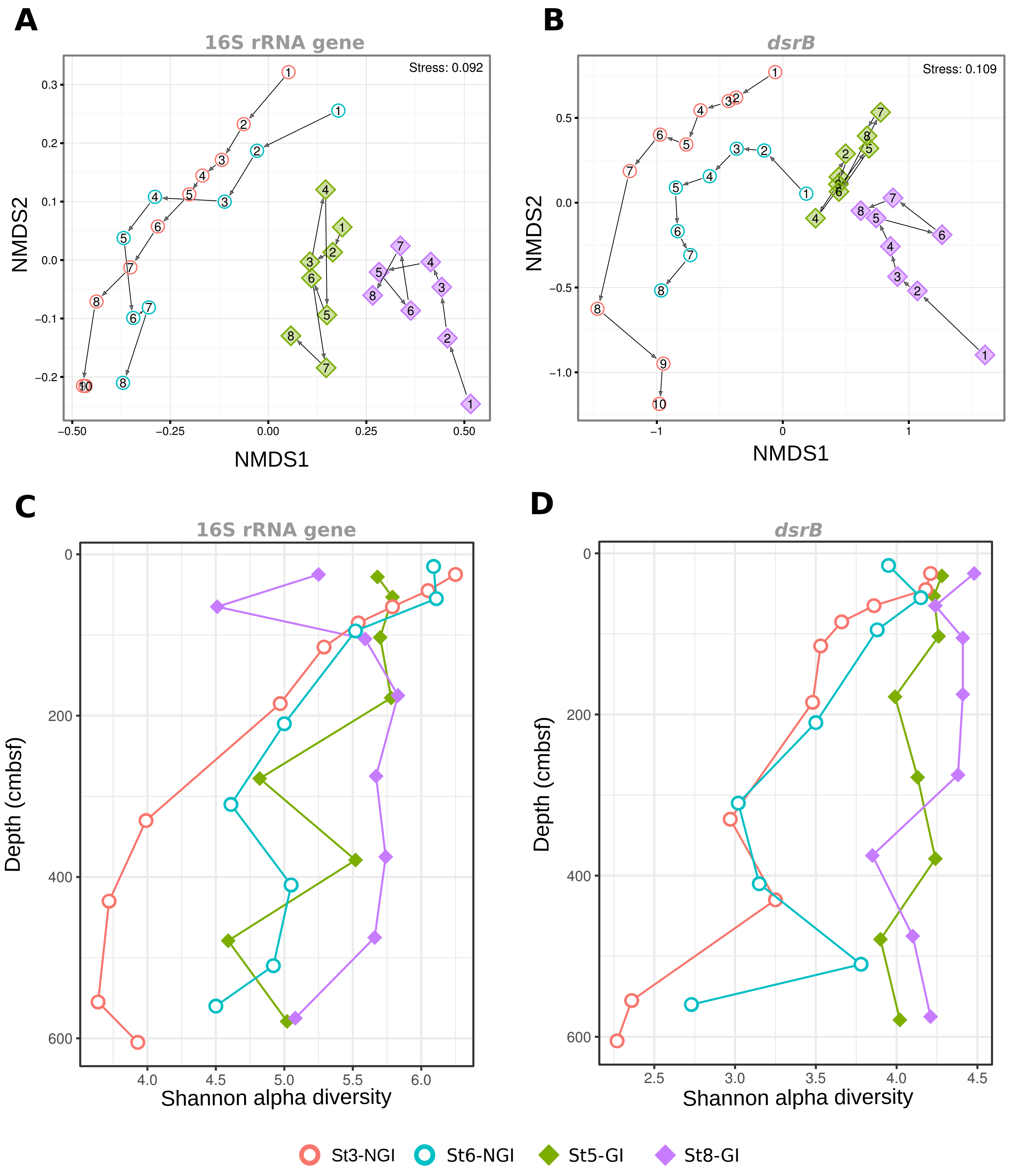


**Supplementary Figure S2. Microbial diversity across non-glacier-influenced (NGI) and glacier-influenced (GI) sediment cores and sediment depth.** A and B, Nonmetric multidimensional scaling ordinations (NMDS) of Bray-Curtis distances between 16S rRNA gene and *dsrB* communities. C and D, Shannon alpha diversity indices of 16S rRNA gene and *dsrB* communities. Colours indicate the sampling station. Numbers indicate the sediment depth according to Supplementary Table 2. St3-NGI and St6-NGI, Non-glacier-influenced stations 3 and 6. St5-GI and St8-GI, Glacier-influenced stations 5 and 8.

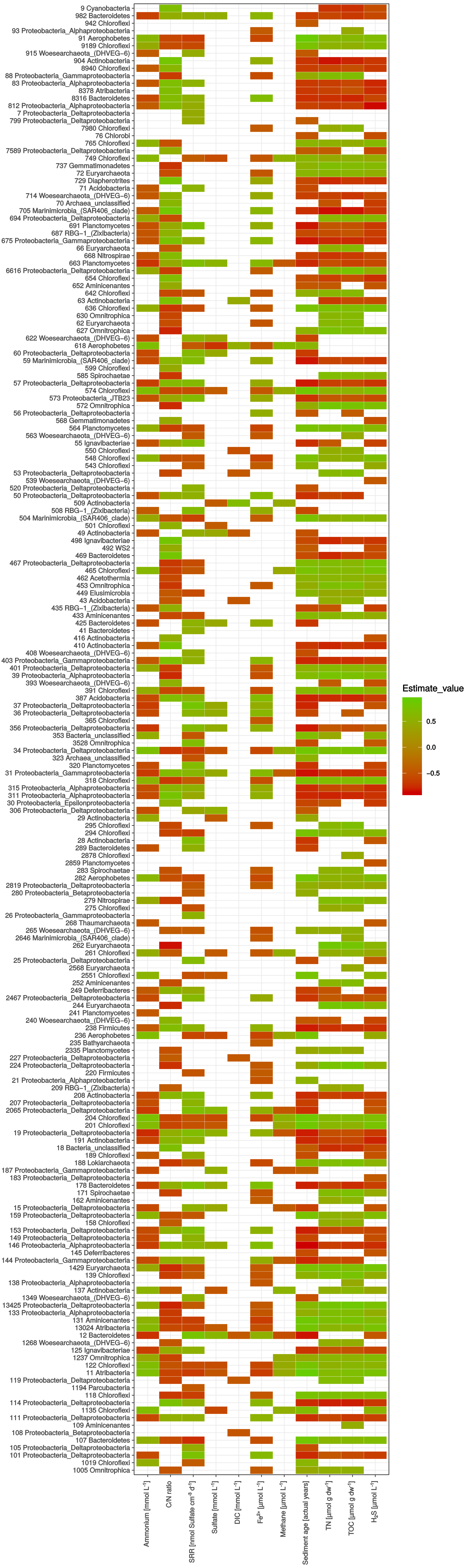


**Supplementary Figure S3. Spearman correlations between relative abundances of 16S rRNA-OTUs and physicochemical parameters.** Indicated are OTUs with significant ($p \leq 0.05$) Spearman correlations to any of the tested parameters.

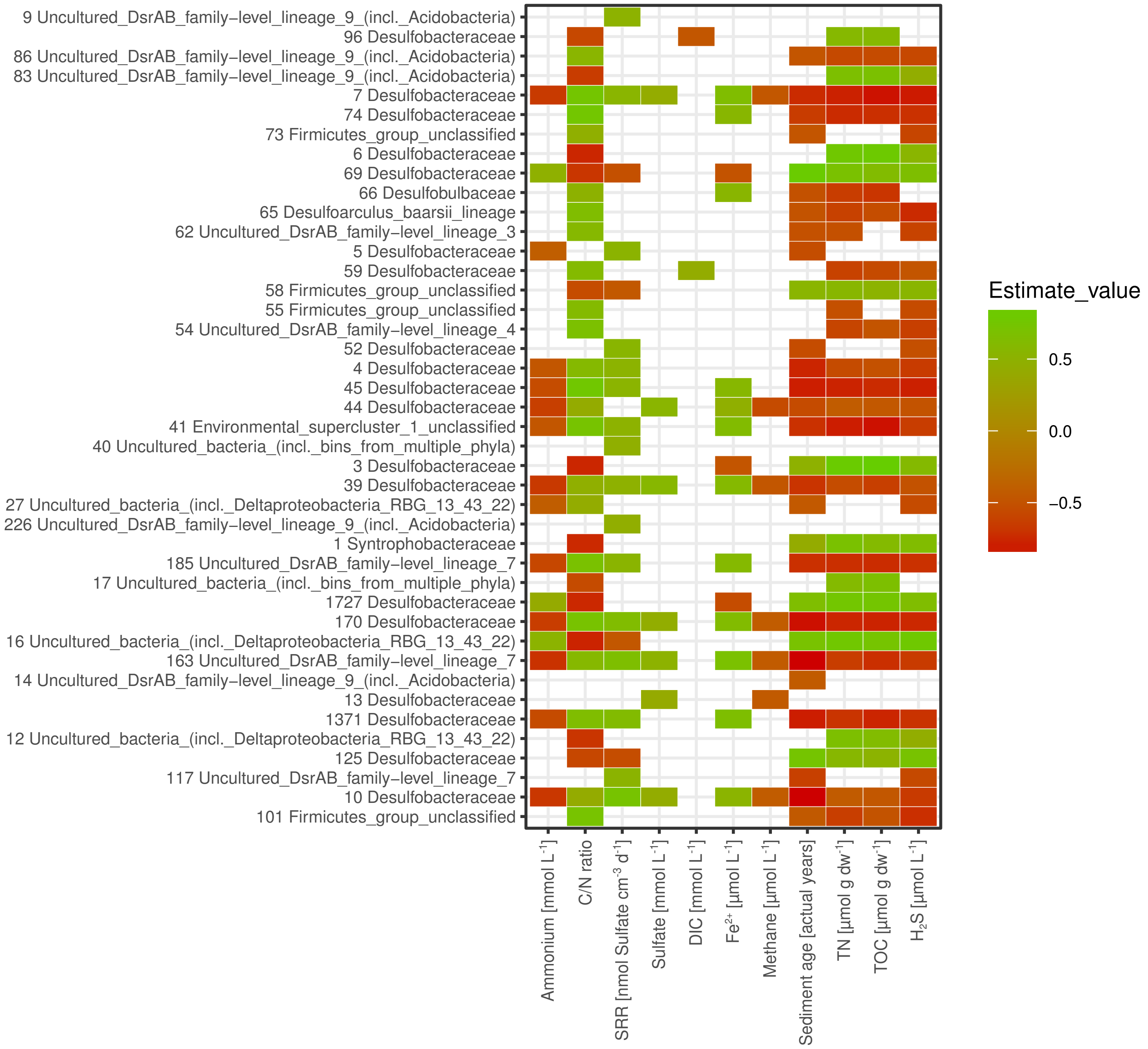


**Supplementary Figure S4. Spearman correlations between relative abundances of *dsrB*-OTUs and physicochemical parameters.** Indicated are OTUs with significant ($p \leq 0.05$) Spearman correlations to any of the tested parameters.

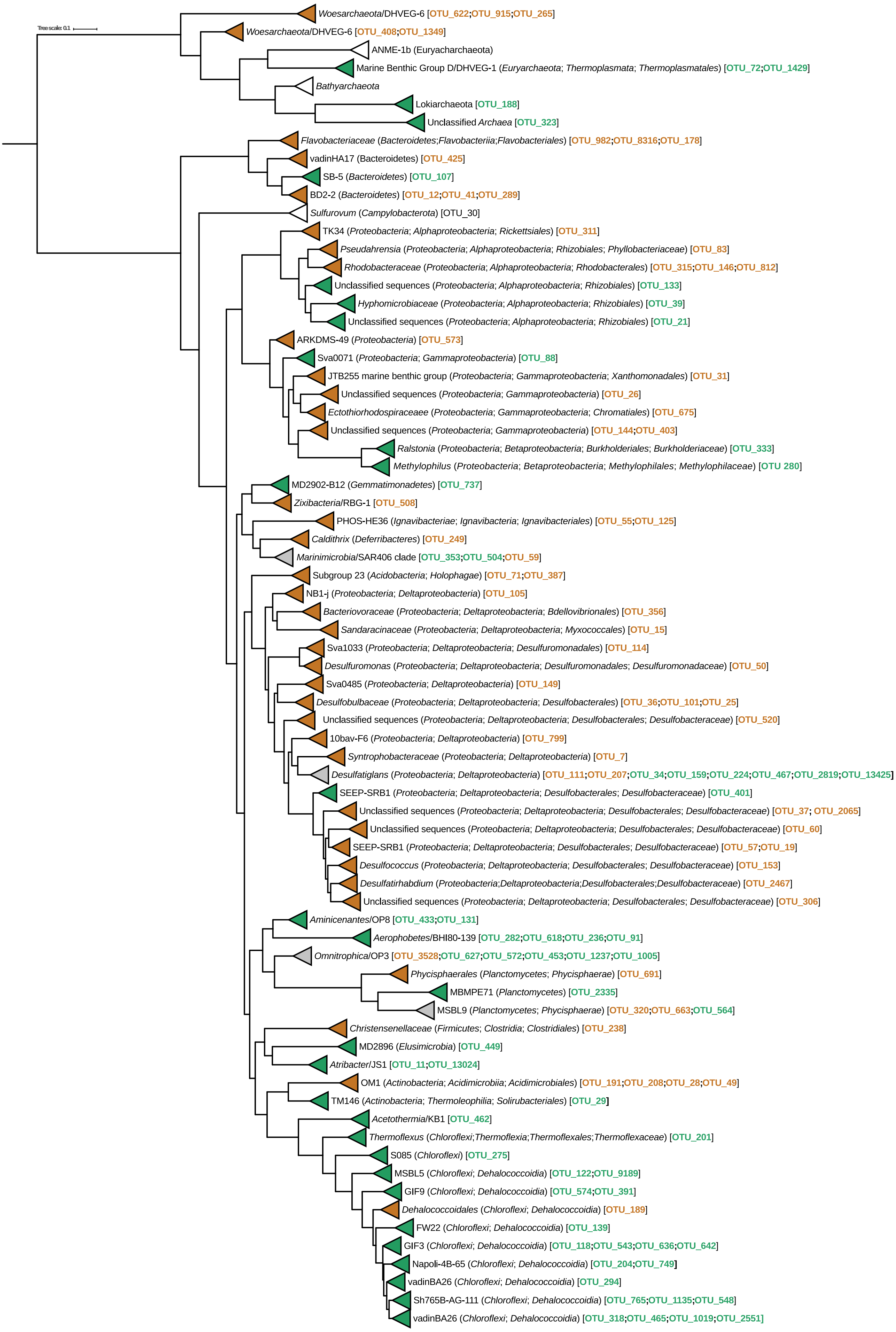


**Supplementary Figure S5. Phylogenetic placement of 16S rRNA-OTUs.** Only OTUs with significant correlations to sulfate reduction rates or sediment age are shown. The reference tree was built from sequences that were closely related to 16S rRNA-OTUs. These were extracted from the SILVA database v.128 (Quast *et al.*, 2013), and used for tree construction with FastTree (Price *et al.*, 2010). 16S rRNA-OTUs were aligned to the SILVA database using the SINA aligner (Pruesse *et al.*, 2012), and placed into the reference tree using the EPA algorithm (Berger *et al.*, 2011) in RAxML (Stamatakis, 2014). The placement tree was visualized with iTOL (Letunic and Bork, 2007). OTUs that are colored in orange and green were significantly correlated to sulfate reduction rates (Figure 3) and sediment age (Figure 4), respectively. Clades that contained only OTUs with significant correlations to either sulfate reduction rates or sediment age are colored accordingly. Gray clades contain OTUs with correlations to sulfate reduction rates and OTUs with correlations to sediment age.

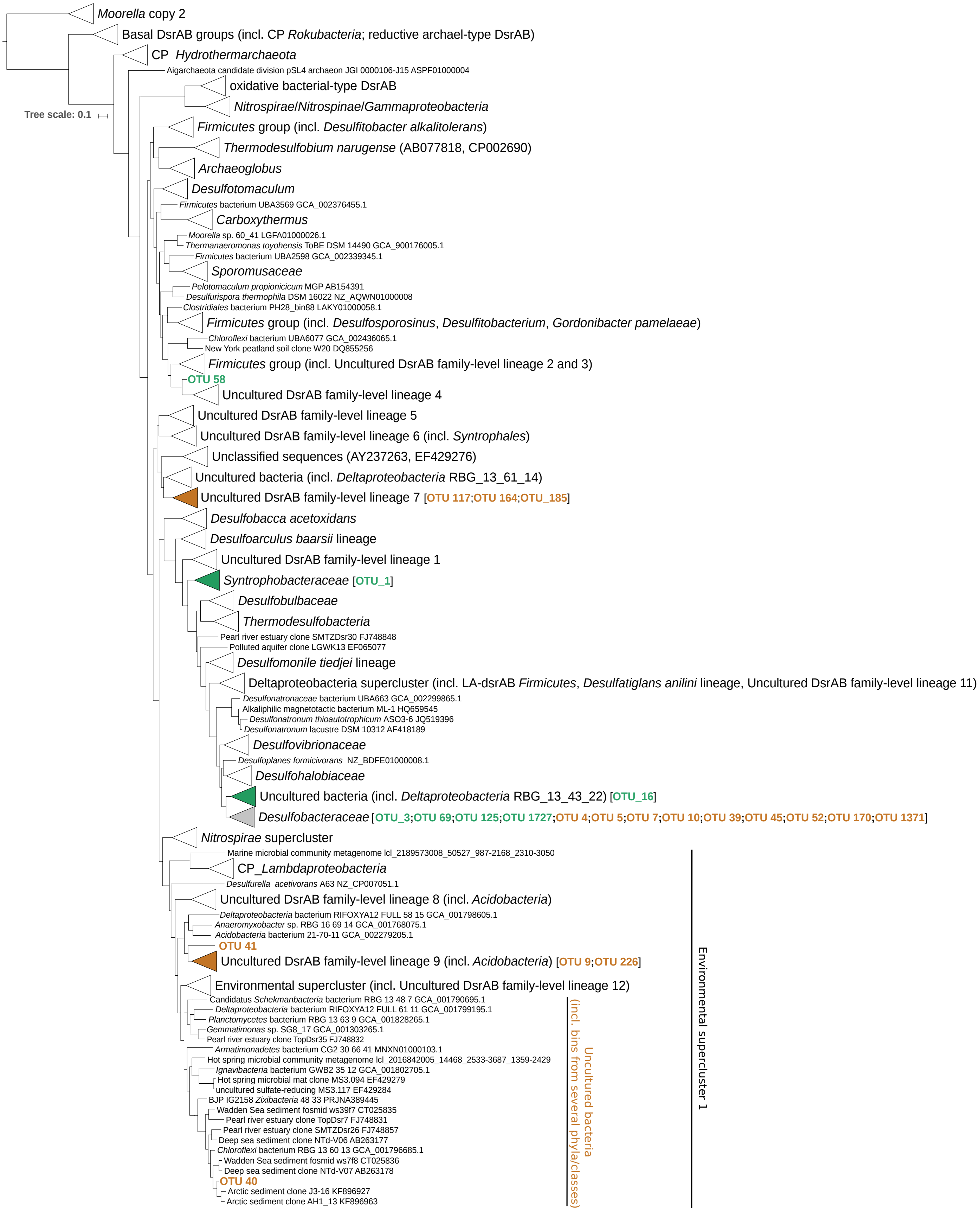


**Supplementary Figure S6. Phylogenetic placement of dsrB-OTUs.** Only OTUs with significant correlations to sulfate reduction rates or sediment age are shown. The reference tree was built from an extended reference alignment that contained DsrAB sequences from Müller et al., 2015 and novel DsrAB sequences from recent metagenomic surveys (Anantharaman et al., 2017; Parks et al., 2017). The reference tree was constructed with FastTree (Price et al., 2010). dsrB-OTUs were aligned to the extended reference alignment using MAFFT (Katoh et al., 2002), and added to the reference tree using the EPA algorithm (Berger et al., 2011) in RAxML (Stamatakis, 2014). The placement tree was visualized with iTOL (Letunic and Bork, 2007). OTUs that are colored in orange and green were significantly correlated to sulfate reduction rates (Figure 5) and sediment age (Figure 5), respectively. Clades that contained only OTUs with significant correlations to either sulfate reduction rates or sediment age are colored accordingly. Gray clades contain OTUs with correlations to sulfate reduction rates and OTUs with correlations to sediment age.

**Supplementary Table S1. Raw data of radiocarbon ages and output of age-depth model of stations St3-NGI, St6-NGI and St5-GI.**

| Core | LabCode | Material | Depth (cm) | 14C Age (yrs BP) | Error (yrs) | Calibrated age range (2 σ) From (cal yrs BP) | To (cal yrs BP) | Modelled age (cal yrs BP) | Error (yrs) |
|---|---|---|---|---|---|---|---|---|---|
| SA13-ST3-20G | AAR20067 | Mollusk fragment | 163.5 | 2362 | 25 | 2083 | 1896 | 2048 | 43 |
| | AAR20068 | Mollusk | 346 | 6446 | 28 | 7038 | 6835 | 6940 | 49 |
| | AAR20069 | Mollusk | 435 | 9033 | 39 | 9875 | 9560 | 9617 | 55 |
| | AAR20070 | Mollusk fragment | 514 | 9896 | 39 | 11020 | 10700 | 10921 | 79 |
| SA13-ST5-30G | AAR20955 | Mollusk | 25 | 652 | 25 | 386 | 250 | 265 | 42 |
| | AAR20956 | Mollusk | 539 | 815 | 25 | 505 | 403 | 457 | 26 |
| SA13-ST6-40G | AAR21687 | Marine macroalgae | 50 | 1372 | 24 | 986 | 840 | 919 | 32 |
| | AAR21690 | Marine macroalgae | 173.5 | 2668 | 28 | 2452 | 2294 | 2362 | 43 |
| | AAR21691 | Marine macroalgae | 196 | 3356 | 30 | 3326 | 3120 | 3218 | 54 |
| | AAR21692 | Marine macroalgae | 260 | 4458 | 40 | 4787 | 4516 | 4647 | 75 |

**Supplementary Table S2. Sample numbering and sediment depth of the four sediment cores.**

| Coring station | Sample numbering | Sediment depth (cmbsf) |
|---|---|---|
| Station 3 | 1 | 25 |
| Station 3 | 2 | 45 |
| Station 3 | 3 | 65 |
| Station 3 | 4 | 85 |
| Station 3 | 5 | 115 |
| Station 3 | 6 | 185 |
| Station 3 | 7 | 330 |
| Station 3 | 8 | 430 |
| Station 3 | 9 | 555 |
| Station 3 | 10 | 605 |
| Station 5 | 1 | 28 |
| Station 5 | 2 | 53 |
| Station 5 | 3 | 103 |
| Station 5 | 4 | 178 |
| Station 5 | 5 | 278 |
| Station 5 | 6 | 379 |
| Station 5 | 7 | 479 |
| Station 5 | 8 | 579 |
| Station 6 | 1 | 15 |
| Station 6 | 2 | 55 |
| Station 6 | 3 | 95 |
| Station 6 | 4 | 210 |
| Station 6 | 5 | 310 |
| Station 6 | 6 | 410 |
| Station 6 | 7 | 510 |
| Station 6 | 8 | 560 |
| Station 8 | 1 | 25 |
| Station 8 | 2 | 65 |
| Station 8 | 3 | 105 |
| Station 8 | 4 | 175 |
| Station 8 | 5 | 275 |
| Station 8 | 6 | 375 |
| Station 8 | 7 | 475 |
| Station 8 | 8 | 575 |

**Supplementary Table S3. dsrB-targeted primer sets that were used in this study**

| Name | Target | Sequence (5'-3') | Reference |
|---|---|---|---|
| DSR1762F1 | dsrB | CAYACCCAGGGNTGG | Steger et all., 2011 |
| DSR1762F4 | dsrB | CACACDCAGGGNTGG | Steger et all., 2011 |
| DSR1762F2 | dsrB | CAYACBCAAGGNTGG | Steger et all., 2011 |
| DSR1762F3 | dsrB | CATACDCAGGGHTGG | Steger et all., 2011 |
| DSR1762F5 | dsrB | CATACHCAGGGNTAY | Steger et all., 2011 |
| DSR1762F6 | dsrB | CACACSCAGGGKTAY | Pelikan et al., 2016 |
| DSR1762F7 | dsrB | CACACBCAGGGMTAC | Pelikan et al., 2016 |
| DSR1762F8 | dsrB | CACACHCAGGGCTAT | Pelikan et al., 2016 |
| DSR1762F9 | dsrB | CACACCCAGGGWTTC | Pelikan et al., 2016 |
| DSR1762F10 | dsrB | CAYACACAAGGATGG | Pelikan et al., 2016 |
| DSR2107R1 | dsrB | CAGTTDCCRCARTACAT | Pelikan et al., 2016 |
| DSR2107R2 | dsrB | CAGTTACCRCAGAACAT | Pelikan et al., 2016 |
| DSR2107R3 | dsrB | CAGTTGSCGCAGAACAT | Pelikan et al., 2016 |
| DSR2107R4 | dsrB | CAGTTYCCGCAGAACAT | Pelikan et al., 2016 |
| DSR2107R5 | dsrB | CAGTTKCCACAGAACAT | Pelikan et al., 2016 |
| DSR2107R6 | dsrB | CAATTWCCACAGAACAT | Pelikan et al., 2016 |
| DSR2107R7 | dsrB | CAGTTGCCGCAAAACAT | Pelikan et al., 2016 |
| DSR2107R8 | dsrB | CAGGCACCGCAGAACAT | Pelikan et al., 2016 |
| DSR2107R9 | dsrB | CAGTTAGCGCACATCAT | Pelikan et al., 2016 |
| DSR2107R10 | dsrB | CAATTGGCACAGTACAT | Pelikan et al., 2016 |
| DSR2107R11 | dsrB | CAATTTCCGCAGTACAT | Pelikan et al., 2016 |
| DSR2107R12 | dsrB | CAATTGCCGCAGAACAT | Pelikan et al., 2016 |
| DSR2107R13 | dsrB | CAGTTGGCACAATACAT | Pelikan et al., 2016 |

## Rerences

Anantharaman, K., Hausmann, B., Jungbluth, S. P., Kantor, R. S., Lavy, A., Warren, L. A., et al. (2018). Expanded diversity of microbial groups that shape the dissimilatory sulfur cycle. ISME J. 12, 1715–1728.

Berger, S. A., Krompass, D., and Stamatakis, A. (2011). Performance, accuracy, and Web server for evolutionary placement of short sequence reads under maximum likelihood. Syst. Biol. 60, 291–302.

Katoh, K., Misawa, K., Kuma, K.-I., and Miyata, T. (2002). MAFFT: a novel method for rapid multiple sequence alignment based on fast Fourier transform. Nucleic Acids Res. 30, 3059–3066.

Letunic, I., and Bork, P. (2007). Interactive Tree Of Life (iTOL): an online tool for phylogenetic tree display and annotation. Bioinformatics 23, 127–128.

Parks, D. H., Rinke, C., Chuvochina, M., Chaumeil, P.-A., Woodcroft, B. J., Evans, P. N., et al. (2017). Recovery of nearly 8,000 metagenome-assembled genomes substantially expands the tree of life. Nat Microbiol 2, 1533–1542.

Pelikan, C., Herbold, C. W., Hausmann, B., Müller, A. L., Pester, M., and Loy, A. (2016). Diversity analysis of sulfite- and sulfate-reducing microorganisms by multiplex dsrA and dsrB amplicon sequencing using new primers and mock community-optimized bioinformatics. Environ. Microbiol. 18, 2994–3009.

Price, M. N., Dehal, P. S., and Arkin, A. P. (2010). FastTree 2 – Approximately Maximum-Likelihood Trees for Large Alignments. PLoS One 5, e9490.

Pruesse, E., Peplies, J., and Glöckner, F. O. (2012). SINA: accurate high-throughput multiple sequence alignment of ribosomal RNA genes. Bioinformatics 28, 1823–1829.

Quast, C., Pruesse, E., Yilmaz, P., Gerken, J., Schweer, T., Yarza, P., et al. (2013). The SILVA ribosomal RNA gene database project: improved data processing and web-based tools. Nucleic Acids Res. 41, D590–6.

Stamatakis, A. (2014). RAxML version 8: a tool for phylogenetic analysis and post-analysis of large phylogenies. Bioinformatics 30, 1312–1313.

Steger, D., Wentrup, C., Braunegger, C., Deevong, P., Hofer, M., Richter, A., et al. (2011) Microorganisms with novel dissimilatory (bi)sulfite reductase genes are widespread and part of the core microbiota in low-sulfate peatlands. Appl Environ Microbiol 77: 1231–1242.
